# When is a mutation not a mutation: the case of the c.594-2A>C splice variant in a woman harbouring another BRCA1 mutation in trans 

**DOI:** 10.1186/s13053-015-0045-y

**Published:** 2016-02-16

**Authors:** Michelle Wong-Brown, Mary McPhillips, Margaret Gleeson, Allan D. Spigelman, Cliff J. Meldrum, Susan Dooley, Rodney J. Scott

**Affiliations:** Information Based Medicine Program, Hunter Medical Research Institute, Kookaburra Circuit, Newcastle, NSW 2305 Australia; School of Biomedical Sciences and Pharmacy, Faculty of Medicine and Health, University of Newcastle, Callaghan, NSW 2308 Australia; Division of Molecular Medicine, Pathology North, John Hunter Hospital, Lookout Road, Newcastle, NSW 2305 Australia; Hunter Family Cancer Service, Hunter New England Health District, Newcastle, NSW 2300 Australia; UNSW Medicine, St. Vincent’s Hospital Clinical School, Sydney, Sydney, NSW 2010 Australia

**Keywords:** BRCA1 mutations, Bi-allelic, Isoforms, Splice site

## Abstract

Since the identification of *BRCA1* there has only ever been described two bi-allelic mutation carriers, one of whom was subsequently shown to be a mono-allelic carrier. The second patient diagnosed with two BRCA1 mutations appears to be accurate but there remain some questions about the missense variant identified in that patient.

In this report we have identified a woman who is a bi-allelic mutation carrier of BRCA1 and provide an explanation as to why this patient has a phenotype very similar to that of any mono-allelic mutation carrier. The splice variant identified in this patient appears to be associated with the up-regulation of a BRCA1 splice variant that rescues the lethality of being a double mutant.

The consequences of the findings of this report may have implications for mutation interpretation and that could serve as a model for not only BRCA1 but also for other autosomal dominant disorders that are considered as being embryonically lethal.

## Introduction

There have been a considerable number of mutations identified in *BRCA1* since its identification in 1994 [1]. During this time in excess of 15,000 *BRCA1* variants have been submitted to the National Human Genome Research Institute (NHGRI)-supported Breast Cancer Information Core (http://research.nhgri.nih.gov/bic/) database. Patients harbouring a deleterious change in *BRCA1* have a significantly increased risk of developing breast and or ovarian cancer [2]. Nonsense mutations represent the majority of unequivocal deleterious mutations in *BRCA1* [3]. Currently, much attention has been focused on insertion/deletion mutations, missense mutations and intron-exon boundary changes since these are more difficult to classify with respect to their pathogenicity. Recent advances in the classification of these types of alteration via the development of algorithms have significantly helped in defining pathogenicity, but it remains difficult to accurately and rapidly classify all of the variants that have been identified to date [4].

Since the identification of *BRCA1* much has been learned about its function, but one of the earliest findings was the paucity of bi-allelic mutations in founder populations [5, 6] and in mouse models of disease, where colonies of heterozygote *Brca1* mice never produced any viable homozygous mutant offspring [7]. Collectively, this information implied that bi-allelic *BRCA1* mutations were embryonically lethal. This is in contrast to bi-allelic *BRCA2* mutation carriers who do survive but invariably develop Fanconi anemia [8].

There have been two reports of patients with bi-allelic mutation in *BRCA1* [6, 9]. The report by Boyd et al. [9] of a woman who was apparently a homozygous carrier of a c.2800delAA has since been shown to be a PCR artefact where preferential amplification on one allele masked the presence of the other [10]. The more recent report by Domchek et al. [6] of a developmentally delayed patient with short stature and an ovarian cancer diagnosed at 28 years of age, an unusual age even for a *BRCA1* mutation carrier, appears to be the first confirmed report of a patient with two *BRCA1* mutations *in trans*. One mutation in this patient is unequivocally causative, a c.2576delC change residing in exon 11 and a missense change c.5207 T > C (Val1736Ala) located in exon 20. The Val1736Ala missense variant has been subjected to an extensive battery of tests that includes *in silico* analysis in conjunction with segregation and functional analysis, which clearly indicate its pathogenicity. However, there remains some questions about the pathogenicity of Val1736Ala, as logistic regression modelling suggests otherwise [11] and it is not known what, if any, residual function can be associated with the variant. This raises the possibility that the missense variant may retain function and that other factors may be involved that are not related to BRCA1 that may have led to her early demise. The more recent report by Sawyer et al. [12] highlighted a patient with the same developmental delay and diagnosis of breast cancer at 23 years of age. This patient harboured one unequivocally causative mutation, c.594_597del (p.Ser198Argfs*35) and another missense mutation on exon 18 (c.5095C>T; p.Arg1699Trp) that has previously been reported to be pathogenic and confers increased risk to breast and ovarian cancers [13]. This patient, however, was deduced to have a subtype of FA.

We herein report on a woman who developed breast cancer at an early age with no developmental delay who was referred to our laboratory for genetic testing and subsequent follow-up studies since she was shown to harbouran unequivocal BRCA1 mutation and a splice site mutation *in trans*, both of which are predicted to result in the absence of functional full-length wildtype BRCA1.

## Methods

This study was approved by the institutional review board of the Hunter New England Health service.

### DNA Extraction

DNA was extracted from peripheral whole blood by salt extraction.

### BRCA1 Mutation Analysis

The entire coding sequence including the intron-exon boundaries of BRCA1 was amplified using primer sequences design in the laboratory. The PCR reactions were treated with ExoSAP (Thermo Fisher Scientific) prior to being bi-directionally sequenced with the BigDye^®^ Terminator v3.1 Cycle Sequencing Kit and capillary-electrophoretically separated using an ABI3730 DNA Analyzer automated sequencer (Applied Biosystems). Sequencing traces were analysed using Mutation Surveyor v3.24 (SoftGenetics^®^). Mutations were described using the nomenclature guidelines of the Human Genome Variation Society (http://www.hgvs.org). The DNA sequence numbering is based on the cDNA sequences for BRCA1 (NM_007294.3).

### RNA Extraction

Lymphocytes isolated from the patients were infected with Epstein-Barr virus (EBV) to establish the immortal lymphoblastoid cell lines (LCLs). These LCLs were maintained in RPMI media (Thermo Scientific) supplemented with 10 % fetal calf serum (Sigma-Aldrich) in an incubator set to 37 °C and 5 % CO_2_. Puromycin (InvivoGen) was added to the EBV cells to prevent RNA degradation by nonsense-mediated decay (NMD).

Total RNA was isolated from cells of carrier patients using Trizol® (Invitrogen^TM^) according to the protocol provided by the manufacturer. RNA was reverse transcribed to cDNA using SuperScript™ II reverse transcriptase (Invitrogen™) according to the protocol provided by the manufacturer.

### Sequencing of *BRCA1* isoforms

Primers were designed to flank the region of interest (Invitrogen™). One forward primer (5′-TGAGAACTCTGAGGACAAAGCA-3′) is located in exon 8 and the other (5′-CTGAAGATACCGTTAATAAGGCA-3′) is located in exon 9. The reverse primer (5′-CCCTGATACTTTCTGGATGC) is located in exon 11. Polymerase chain reaction (PCR) was conducted under the following cycling conditions: denaturation at 95 °C for 5 min; 32 cycles of 95 °C for 30 s, 59 °C for 40 s and 72 °C for 40, and extension at 72 °C for 10 min. The PCR products were then size-separated by electrophoresis on a 2 % agarose gel.

Direct sequence analysis was performed using the BigDye® Terminator v3.1 Cycle Sequencing Kit (Applied Biosystems) and size separated by capillary-electrophoresis using an ABI3730 DNA Analyzer (Applied Biosystems). Sequencing traces were analysed using Mutation Surveyor v3.24 (SoftGenetics®).

### Real-time analysis of *BRCA1* isoforms

Real-time PCR was performed using SYBR® Green PCR amplification on an Applied Biosystems ABI PRISM® 7900HT Sequence Detection System (SDS). A 12.5 μl reaction volume was set up, which included 6.25 μl SYBR® Green PCR MasterMix, 0.25 μl B-actin (housekeeping gene) or custom *BRCA1* forward and reverse primers (5 μM) (Table 1), and 0.625 μl reverse transcription product from the patient and two controls. All reactions were run in triplicate. The reactions were incubated at 50 °C for 2 min and 95 °C for 10 min, followed by 40 cycles of 95 °C for 15 s and 60 °C for 1 min. The results were analysed using the SDS software.

**Table 1:** Sequences of forward and reverse primers used for the detection of expression of *BRCA1* isoforms by RT-PCR

Isoform	Forward sequence	Reverse sequence
Wildtype *BRCA1* (does not cover truncation in exon 11)	5′-GCAACTTATTGCAGTGTGGG-3′	5′-ACAAGCAGCCTTTTTTGCAG-3′
Exon 10 deleted (Δ10)	5′- GCAACTTATTGCAGCTGCTTG-3′	5′- AGCTGCACGCTTCTCAGTG-3′
Exon 9 and 10 deleted (Δ9/10)	5′- GTCTGTCTACATTGAATTGGCTG-3′	5′- AGCTGCACGCTTCTCAGTG-3′
Exon 11q deleted (Δ11q) [12]	5′-CCAACTCTCTAACCTTGGAACTGTG-3′	5′-GATGACCTTTCCACTCCTGGTTC-3′

## Results

**Fig. 1:**
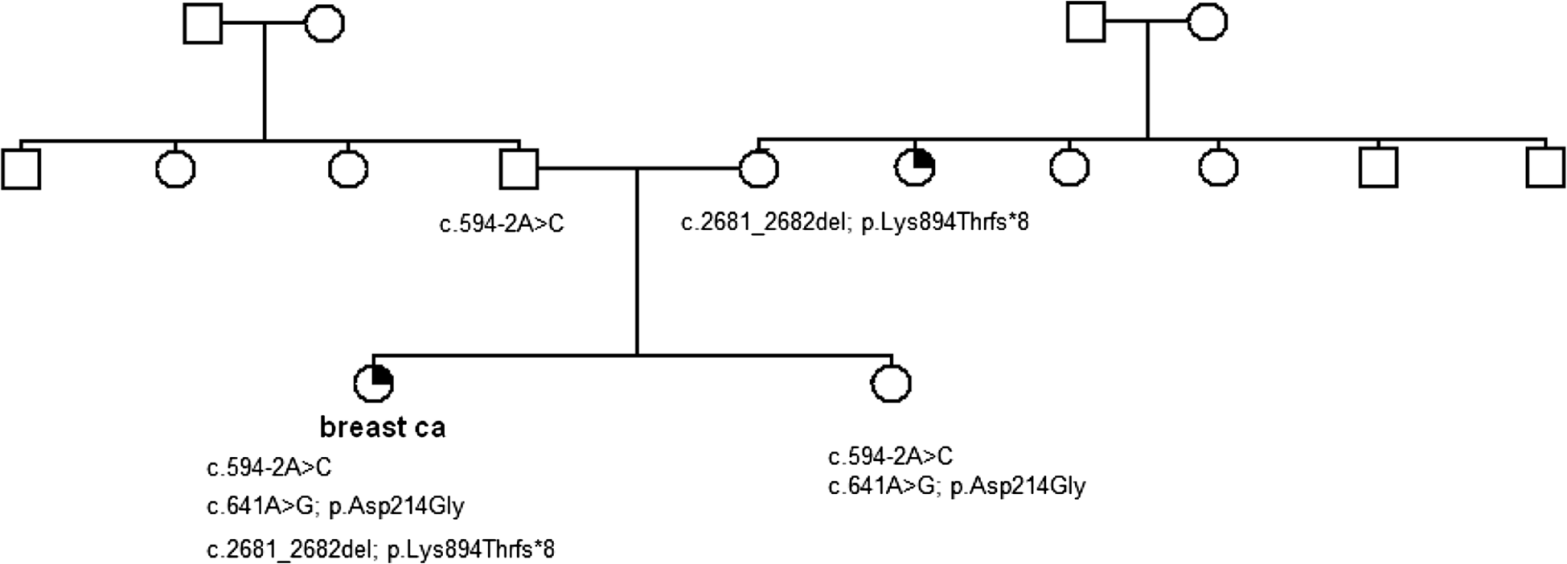
Pedigree of the family. Mutations present in this family are stated below the symbols of the individuals tested

The patient presented with breast cancer at 30 years of age without any immediate family history (pedigree shown in Fig. 1). The tumour was defined as being estrogen, progesterone and HER2 receptor negative, and thus suggesting that due to her young age she was likely to have a genetic predisposition to early onset disease.

**Fig. 2:**
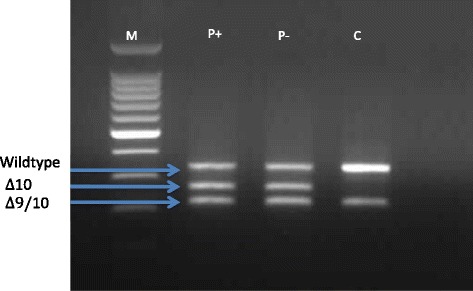
Analysis of the BRCA1 mutation c.594-2 A > C by agarose gel electrophoresis. PCR products amplified using primers specific for exon 8 and exon 11 of the BRCA1 gene. P is the patient sample and C is the control sample. Puromycin treated (P+) and puromycin non-treated (P −) are indicated in the figure. M is the 100 bp DNA ladder (Promega). Three different transcripts are present in the proband (P) representing a wildtype fragment, a fragment lacking exon 10 and a fragment representing the Δ9/10 isoform Only the wildtype fragment and the Δ9/10 isoform are present in the control sample (C)

Genetic testing of DNA derived from her peripheral blood lymphocytes revealed a frameshift mutation in exon 11 of BRCA1 (c.2681_2682delAA) and a splice site change located in intron 9 that results in the absence of exon 10. This splice site change, c.594-2A > C, is predicted to be a splice site mutation that causes a frameshift, resulting in a premature stop codon). Genetic testing of the mother revealed that she carried the c.2681_2682delAA mutation and the father the c.594-2A > C splice site variant. The *BRCA1* exon 11 mutation is clearly pathogenic and is associated with an increased risk of early onset breast and/or ovarian cancer. The presence of two potentially pathogenic mutations *in trans* observed in a single individual appears to be contrary to the evidence provided by animal models and founder populations where *BRCA1* mutations *in trans* are either embryonically lethal or unreported [6, 7]. The presence of a splice site mutation does not automatically infer that splicing is affected, so to ensure that splicing was indeed interrupted we designed a series of primers to interrogate the expression of the allele that carried the splice site variant. Primers located in exon 8 and exon 11 were used to identify which transcripts were present in the cDNA of the proband compared to a healthy control subject. The primer used in exon 11 was 5′ of the c.2681_2682del mutation and as such is referred to as the “wildtype” allele. The results revealed the presence of three transcripts, the “wildtype” transcript, the exon 10-deleted (Δ10) transcript and a commonly occurring isoform with exons 9 and 10 deleted (Δ9/10) transcript (Fig. 2). In the healthy control subject only the wildtype and Δ9/10 transcript can be identified, confirming the specificity of the exon 10 deletion in the proband.

The paternal allele carries a c.641G polymorphism in exon 10 (Gly214) which is in linkage disequilibrium with the c.594-2A > C mutation. If read-through of the splicing variant was occurring, the allele produced would lead to the translation of a functional BRCA protein. To determine whether this allele was expressed, a PCR was performed using primers which annealed to exon 9 and exon 11 (5′ of the maternal mutation). The amplified fragment would exclude the 9/10del isoform but would include exon 10 of the maternal allele as well as the paternal allele if present. If this fragment was heterozygous for the 641A > G polymorphism it would indicate that exon 10 of the paternal allele was present, indicating that the c.594-2A > C mutation is not fully penetrant.

Our results revealed that only the maternal wildtype allele was expressed and that no paternal allele could be detected (Fig. 2). This result confirmed that the c.594-2A > C did affect the expression of the paternal allele, thus the functional BRCA1 (Δ9/10) trranscript must be produced from an alternatively-spliced isoform.

**Fig. 3:**
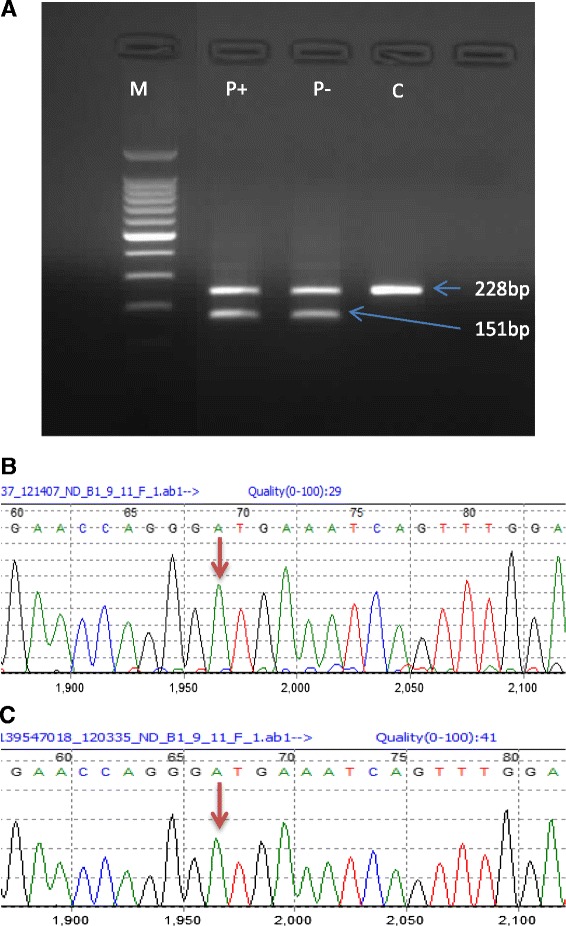
Analysis of the BRCA1 variant c.641A>G. **a** Agarose gel showing PCR products amplified using primers specific for exon 9 and exon 11 of the BRCA1 gene. P is the patient sample while C is the control sample. Puromycin treated (+) and puromycin non-treated (-) are indicated in the figure. M is the 100bp ladder size marker (Promega). The band at 228bp represents the fragment amplified from exon 9 to exon 11 and includes exon 10. The band at 151bp represents the fragment lacking exon 10. **b** Sequencing analysis of the 228bp band for the patient indicates the presence of the c.641A allele (arrow), but not c.641G. **c** Sequencing of the 228bp band for the control sample indicates the presence of the c.641A allele

**Fig. 4:**
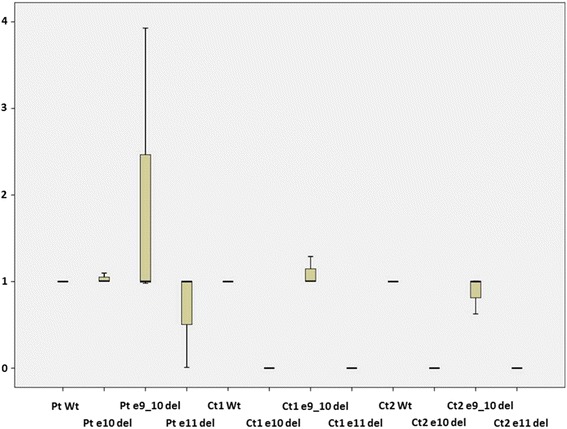
Relative expression (2-ΔΔCT) of each of the BRCA1 isoforms compared to the wildtype BRCA1 in patient and controls. The BRCA1 Δ10 and Δ11q isoforms are not present in any of the two controls

Since the maternal and paternal alleles are not expressed as a result of the respective mutations they carry, the question arises as to what is occurring in the proband that has allowed her to survive and present with a phenotype that is similar, to a women harbouring a single deleterious change in *BRCA1*. We aimed to determine whether one or more isoforms of BRCA1 could rescue the phenotype such that a functional BRCA1 transcript was produced but not of full length (Fig. 3). For this analysis we used real-time polymerase chain reaction (RT-PCR) to quantify the levels of transcripts present in the proband and a series of control subjects (see Fig. 4). Primers were designed to specifically capture the expression levels of the Δ10 transcript, as well as the Δ9/10 isoform and another functional exon 11q-deleted (Δ11q) isoform [14]. Both the Δ9/10 isoform and the Δ11q isoforms are in-frame and have potential residual functional activity. We compared the results from the proband against a normal control and a second breast cancer patient who harboured a mutation in one allele that resulted in the deletion of exon 10 (c.581C > T;p.Ala194Val). The results revealed that in the control subject expression of the Δ9/10 isoform is detectable at approximately the same level as the wildtype BRCA1 transcript. There is also expression of the Δ9/10 isoform that is similar to wildtype BRCA1 expression levels in the breast cancer patient control (heterozygous mutation carrier). In both of these subjects, the Δ11q isoform and the Δ10 isoform (specific to the proband) were not detectable. The Δ9/10 isoform was substantially upregulated by about 2.5-fold relative to the levels of the BRCA1 “wildtype” transcript in the proband. The Δ10 transcript was observed to be expressed at approximately similar levels to the “wildtype” transcript, and we also showed the expression of the Δ11q isoform. This collective observations suggest that the Δ9/10 isoform and the Δ11q isoform is upregulated in the proband compared to that observed in the control subject and another breast cancer patient control.

## Discussion

There is a paucity of patients harbouring BRCA1 mutations *in trans*, which is considered to be due to the embryonically-lethal nature of these events. Epidemiological evidence in founder populations and animal models support this view and to date only one example of a patient harbouring two mutations *in trans* has been reported [6]. Even though the report from Domchek et al. (2012) is comprehensive in its assessment of the missense change there remain some questions about the reported patient. Given the developmental delay, short stature and microcephaly observed in the patient reported by Domchek et al. (2012), it remains to be determined if there were other genetic alterations that could give rise to the patient’s severe symptoms. And like this previous report, a more recent one by Sawyer et al. (2015) [12] showed a patient with biallelic BRCA1 mutations *in trans* with a similar phenotype.

Like the splice site mutation in our proband, there is a possibility that the missense Val1736Ala and the Arg1699Trp mutations possibly retain some function, as the two probands harbouring the missense and nonsense mutations *in trans* have a low age of cancer onset even for monoallelic BRCA1 mutation carriers [6]. Nevertheless, these patients harboured an unequivocal BRCA1 mutation and a variation of unknown functional significance that is very likely to be pathogenic. The difference between this patient and the one reported herein is that there appears to be a rescue of the phenotype by virtue of the fact that a functional isoform is upregulated on the allele harbouring the c.594-2A>C variant. The functional isoform is linked to the presence of the intronic splice site mutation adjacent to exon 10. This alteration results in the loss of exon 10 and concomitantly activates a known isoform that appears to be similar in function to the wildtype allele [15]. In support of this, we were fortunate enough to encounter another unrelated patient who harboured a single mutation at the 3’ end of exon 9 (c.581C>T, p.Ala194Val), also predicted to result in alternate splicing.  Interestingly, in this patient it was observed that the Δ9/10 isoform was also upregulated but not to the same extent as that observed in the proband reported herein.  It has been reported that the c.594-2A>C variant is not associated with a highly-penetrant disease profile even though the allele is not expressed in persons who harbour this change [16]. It is likely that as this variant has been detected *in trans* with other unequivocally pathogenic mutations, such as the truncating c.2681_2682del in our patient and the BRCA1 founder variant exon13ins6kb [16], the pathogenicity of this variant has not been until now fully elucidated but has been the subject of investigation [17]. We have unequivocally shown that the BRCA1 c.594-2A>C variant results in a loss of the full-length wildtype allele and the upregulation of a functional BRCA1 isoform. This is, to our knowledge, the first evidence demonstrating the functional activity of the Δ9/10 BRCA1 isoform. Segregation analysis of families harbouring this variant clearly indicates reduced penetrance that is at odds with expression data which unequivocally shows the absence of the allele-specific transcript [18]. In the absence of any other information this would create a conundrum in terms of what it meant to harbour this change. The data from the proband suggests there is a feedback loop associated with the splice site mutation and the expression of the Δ9/10 isoform that results in the rescue of the phenotype, which is consistent with the report from Dosil et al. (2010) who characterised the role of the splice variant c.591C>T in the generation of the Δ9/10 isoform [19]. Taken together, result explains the significantly reduced disease penetrance observed in mono-allelic carriers of the c.594-2A>C variant.

## Conclusion

We have an example of a true biallelic mutation in *BRCA1*, both of which significantly alter the full-length transcripts of BRCA1. However, as a result of upregulation of the Δ9/10 functional isoform rescue of the phenotype occurs. Without the compensation of the Δ9/10 isoform it is likely that either the patient would have not developed in utero or would have had severe mental retardation. This implies that the Δ9/10 isoform is functional and capable of orchestrating most, if not all, of the function of the wildtype allele. Acknowledgement of this finding will make genetic analysis and counselling issues more complex since mutations occurring in region of exons 9 and 10 of *BRCA1* may not result in predicted outcomes based on sequence analysis alone.
