# Correction: When is a mutation not a mutation: the case of the c.594-2A>C splice variant in a woman harbouring another BRCA1 mutation in trans

**DOI:** 10.1186/s13053-022-00228-y

**Published:** 2022-05-30

**Authors:** Michelle Wong-Brown, Mary McPhillips, Margaret Gleeson, Allan D. Spigelman, Cliff J. Meldrum, Susan Dooley, Rodney J. Scott

**Affiliations:** 1 Information Based Medicine Program, Hunter Medical Research Institute, Kookaburra Circuit, Newcastle, NSW 2305 Australia; 2 School of Biomedical Sciences and Pharmacy, Faculty of Medicine and Health, University of Newcastle, Callaghan, NSW 2308 Australia; 3 Division of Molecular Medicine, Pathology North, John Hunter Hospital, Lookout Road, Newcastle, NSW 2305 Australia; 4 Hunter Family Cancer Service, Hunter New England Health District, Newcastle, NSW 2300 Australia; 5 UNSW Medicine, St. Vincent’s Hospital Clinical School, Sydney, Sydney, NSW 2010 Australia

**Correction: Hered Cancer Clin Pract 14, 6 (2016)**

**https://doi.org/10.1186/s13053-015-0045-y**

Following publication of the original article [1], it was identified that on page 4 of 7, in the results section, the exon number should be 9 not 10 as written. We apologize for any inconvenience this may have caused.
